# Cervical ribs in Pakistan: an under-recognized anatomical anomaly

**DOI:** 10.1097/MS9.0000000000005063

**Published:** 2026-04-21

**Authors:** Muhammad Ubaid Ur Rehman, Noor Un Nisa, Rafiullah Hotak

**Affiliations:** a Department of Medicine, Gujranwala Medical College, Gujranwala, Pakistan; b Department of Medicine, Jinnah Sindh Medical University, Karachi, Pakistan; c Nangarhar University Faculty of Medicine, Jalalabad, Afghanistan

*To the Editor*,

Cervical ribs are rare innate anomalies that originate from the seventh cervical vertebra. Their global prevalence ranges between 0.5 and 1%, though regional variation is appreciable. These supernumerary ribs may remain asymptomatic or lead to clinical complications such as thoracic outlet syndrome, resulting in neurovascular compression, pain, paresthesia, numbness, tingling, and arm weakness[1].

In Pakistan, cervical ribs remain the least reported condition. Clinical cases are infrequently documented, primarily when patients present with chronic neck pain or upper limb symptoms unresponsive to routine management. Patients are frequently misdiagnosed and treated for neuropathy or cervical spondylosis. Radiological imaging is essential for diagnosis, particularly MRIs, CT scans, and cervical spine X-rays[2]. Based on limited published case reports from Pakistan, there is a paucity of population-specific prevalence data, little awareness, and little diffusion. As evidenced by limited Pakistani data from Karachi, cervical ribs are uncommon and clinically ambiguous in the local backdrop[3].

In order to prevent unintentional harm to nearby neurovascular tissues, surgical planning places a greater emphasis on precise diagnosis, especially in orthopedic and vascular treatments. More awareness among medical professionals is required to enhance results. Congenital abnormalities such as cervical ribs should be included in training programs and updated diagnostic standards, especially for patients presenting with unexplained cervicobrachial symptoms. Additionally, establishing a national registry for congenital musculoskeletal anomalies could facilitate better data collection, assembly, and research. This article aligns with the TITAN Guidelines on the need for transparency in AI use in healthcare[4].

In conclusion, although rare, cervical ribs jeopardize notable clinical challenges. Enhanced awareness, diagnosis, and reporting in Pakistan can lead to better management strategies and a deeper understanding of their epidemiological significance.
